# Supplementary material for: School food and nutrition environments in Australian primary schools: A scoping review
Source: PLoS One. 2025 Jul 1;20(7):e0327310. doi: 10.1371/journal.pone.0327310 (PMC12212514; doi:10.1371/journal.pone.0327310)
Supplement: S1 Appendix — (DOCX) [file pone.0327310.s002.docx]

**S1 Appendix**

Database: ERIC (via Ebscohost)

|  | **Search Terms** | **Number of references** |
| --- | --- | --- |
| S1 | “primary school*” OR “elementary school*” OR “Primary education” OR “elementary education” OR DE(“Elementary Schools” OR “Primary Education” OR “elementary education”) | 431,866 |
| S2 | Australia* OR “New South Wales” OR Victoria OR “Northern Territory” OR Tasmania OR Queensland OR Brisbane OR Sydney OR Perth OR Melbourne OR Hobart OR Adelaide OR Darwin OR Canberra | 200,298 |
| S3 | Food OR nutrition OR diet* OR eat* OR dine OR dining OR meal* OR breakfast* OR lunch* OR “morning tea*” OR dinner OR snack* OR DE(Food OR “Food Service” OR Nutrition OR Dietetics) | 190,671 |
| S4 | System* OR environment* OR curricul* OR polic* OR program* OR resource* OR intervention* OR tool* OR teach* OR educat* OR market* OR garden* OR canteen* OR tuckshop* OR literacy OR cook* OR skill* OR prepar* OR takeaway OR shop* OR store* OR guideline* OR standard* OR DE(“Nutrition Instruction” OR “Home economics” or Breakfast programs” OR “Lunch Programs” OR Curriculum OR “Curriculum Development” OR “Elementary School Curriculum” OR “School Policy” OR “program design” OR “program development” OR “program implementation” OR “program evaluation” OR “program content” OR “program effectiveness” OR intervention OR education OR gardening OR guidelines OR “school activities” OR “school space” OR “school shops” OR “school location” OR “educational facilities” OR resources OR “educational environment” OR “physical environment” OR “classroom environment” OR “teaching methods” OR “teaching guides” OR “home economics”) | 6,183,345 |
| S5 | S1 AND S2 AND S3 AND S4 | 253 |
|  | Filter applied:  Language: English  Publication type: peer reviewed  Publication date: 2021 -2022 | 19 |
