# Supplementary material for: School food and nutrition environments in Australian primary schools: A scoping review
Source: PLoS One. 2025 Jul 1;20(7):e0327310. doi: 10.1371/journal.pone.0327310 (PMC12212514; doi:10.1371/journal.pone.0327310)
Supplement: Attachment — Submitted filename: Suggestions.doc [file pone.0327310.s003.doc]

| **Editor Comments*** | Author Responses | Page number |
| --- | --- | --- |
| **Introduction**   1. Refine the introductory section to ensure clarity and provide a comprehensive understanding of the study's scope. | **Introduction**  Thank you for highlighting that the study scope is not clear. We have refined the introduction and moved contextual data on the Australian school environment to methods (subsection Context). We believe this makes the scope clearer for the audience. | 3-5 |
| 2. Emphasize the study's importance for child development from physical and mental perspectives. | Further to the above (moving the contextual information to methods), we believe this has also made the impacts on child health and wellbeing clearer. Specifically, the introduction includes the following:  *“Internationally, schools are recognised as a key enabling environment to provide access to nutrition and food as well as education and skills for food and nutrition literacy to optimise growth, development, health and wellbeing [5-7]. For children in primary school where there is less autonomy and control over their food, school environments are therefore critical for developing food agency and the optimisation of dietary intake for health and wellbeing [8].”* | 3 |
| 3. Clearly outline the unique contribution of the research to existing literature. | The following paragraph has been included in the introduction section:  *“Several previous reviews have sought to explore, within the primary school setting, health and/or nutrition interventions [10-15], integration of nutrition education into the curriculum [16], the impact of nutrition policies on the SFNE [17, 18] and the environmental impacts of lunchboxes [19]. However, to the best of the knowledge of the authors, no reviews have simultaneously explored all factors that affect the SFNE.”* | 3 |
| **Methodology**   1. Expand on the research methodology, ensuring transparency in design and execution    - Reviewer 1, Suggestion 1 (strengthen the elaboration of research methodology) | **Methodology**  Further details have been provided throughout the methods section which elaborates on the methods undertaken during the review. | 5-8 |
| 2. Include a detailed explanation of the quality assessment tool, particularly distinctions between high, medium, and low quality levels.    - Reviewer 1, Suggestion 2 (Augment the explanation of the quality assessment tool)    - Reviewer 2, second comment (explain quality tool, to align with Table 3) | The critical appraisal section has been updated as follows:  *“A quality assessment was completed on all articles using the relevant Joanna Briggs Institute checklist [29], appraisals for quasi-experimental, cohort, case-study, cross-sectional and qualitative study types. Mixed methods studies were assessed using both the relevant quantitative and qualitative checklists. Each appraisal tool had between eight and eleven criteria. Articles which received affirmative responses for all applicable criteria were classified as high quality. Qualitative studies (including case studies) with three or more negative or unclear responses, and quantitative studies with more than half negative or unclear responses were deemed low quality. All remaining papers were designated as medium quality.”* | 8 |
| 3. Justify the use of the PRISMA model and ensure its implementation is clearly articulated. | PRISMA is the gold standard for systematic reviews and meta-analyses, the PRISMA-ScR checklist extension has been validated as the gold standard for Scoping Reviews (see: Tricco AC, Lillie E, Zarin W, et al. PRISMA Extension for Scoping Reviews (PRISMA-ScR): Checklist and Explanation. Ann Intern Med. 2018;169(7):467-473).  In the manuscript, the subheadings under the methodologies section have been updated to align to the PRISMA-ScR checklist to provide clarity, and further details have been provided throughout. | 5-8 |
| 4. Address the influence of socio-economic factors on the methodology. | The following has been updated to make the analysis of the socio-economic factors analysis clearer in the methods section:  *“Articles which reported on socio-economic factors (Socio-Economic Indexes for Areas (SEIFA Index), school location/remoteness) were also noted, data extracted, and analysed to independently highlight these factors in the results.”* | 7-8 |
| **Results**   1. Supplement the results with improved visual representations, such as diagrams, tables, or charts.    - Reviewer 1, Suggestion 4 (supplement the visual representation of research results) | **Results**  Figure 2 provides a visual summary of the results. In total this paper includes four tables and two figures for data visualisation. We believe this is sufficient visual representation for this paper. Could you please provide further clarification if you believe something else is required. |  |
| 2. Add clear captions and detailed explanations for all tables and figures, ensuring that abbreviations are defined where necessary.    - Reviewer 2, first comment (revise technical mistakes, e.g. abbreviations under tables) | Thank you for pointing this out. We have reviewed the tables and figures, and included legends were appropriate. | Table 2, Table 4 and Figure 2 |
| 3. Discuss regional disparities in findings and their implications.    - Reviewer 1, Suggestion 3 (deepen the analysis of regional disparities in research findings) | The following has been included under the In-depth review of selected articles and synthesis section:  *“Given that most publications were reporting on schools in NSW and the differences in how schools are supported to undertake food and nutrition work, comparisons across regional areas were not feasible.”* | 12 |
| **Discussion**   1. Expand the discussion to include an in-depth analysis of results, mechanisms, and implications.    - Reviewer 1, Suggestion 6 (enhance the depth and breadth of the discussion section) | **Discussion**  We are seeking some further clarification on what is required. We believe we have already provided an in-depth analysis of the results, mechanisms and implications. For example, the discussion opens with a succinct summary of the results, then discusses the enablers and barriers (i.e. mechanisms) to optimal SFNEs, which was a secondary aim of the review. These fitted into three broad categories (as food and nutrition education, student food consumption, and government and school policies), which are discussed in depth in paragraphs 2 to 4 of the discussion section. The final part of this section discusses the socio-ecological considerations (another aim of the review). Additionally, we have provided a discussion of the implications under the Implications for Policy Makers and Researchers section.  We would also like to highlight that Reviewers 2 and 3 did not make any suggestions for this section. | 46-47 |
| 2. Address the limitations of the study, including socio-economic factors and regional variations.    - Reviewer 1, Suggestion 3 (deepen the analysis of regional disparities in research findings)    - Reviewer 1,Suggestion 5 (elaborate on the research limitations regarding the influence of socio-economic factors) | The limitations around regional and socio-ecological factors has been expanded to include:  “*The second limitation was the limited reporting of analysis of the impacts of geographical and socio-ecological considerations on findings among the selected studies. The majority of publications came from NSW and very few studies considered the socio-ecological setting. If care is taken to interpret for local contexts, the findings of this study are valuable for the development of comprehensive strategies to improve SFNEs outside of NSW. However, strong recommendations cannot be made on socio-ecological aspects, except to note the need for researchers to collect data and report more broadly on these factors in research on SFNE.”* | 47-48 |
| 3. Clarify the innovation and contributions of the study in advancing food and nutrition education.    - Reviewer 1, Suggestion 7 (clarify the innovation and contribution of the research) | The following has been included under the discussion section:  *“To the best of the authors knowledge, this is the first review which synthesises the impacts of internal and external factors on the SFNE, advancing a holistic approach to the study of SFNE in the literature.”* | 41 |
| 4. Discuss potential global applications or comparisons to other regions beyond Australia. | This publication sought to summarise the breadth of literature on SFNE in Australian primary schools. We believe it is beyond the scope of this review to discuss comparisons to other regions at any great length as it would not be possible without increasing the word count significantly. However, we have included a statement about the holistic approach this scoping review has undertaken and its utility in an international context:  *The approach of this scoping review has some utility for global applications. Due to the Australian context, findings here cannot be directly extrapolated to other nations. However, the holistic approach undertaken to conceptualise SFNE is a worthy endeavour for SFNE researchers and policy makers alike internationally.* |  |
| Recommendations and Future Research   - Reviewer 1, Suggestion 9 (Increase the prospectiveness and recommendations of the research)  1. Provide actionable recommendations for improving food and nutrition education in schools. | **Recommendations and Future Research**  Thank you for identifying the opportunity to provide more actionable recommendations. The following sentence has been included under the Implications for Policy Makers and Researchers section:  “*SFNE across Australia have similar components, of which one is FNE. There are some barriers to implementation which would benefit from a targeted approach to building FNE interventions across curricular*.” |  |
| 2. Suggest future research directions to address gaps identified in this study, such as the socio-economic and regional disparities. | The following sentence has been updated to include regional disparities  “*A key implication of this review for researchers highlights the need to consider the interconnected nature of SFNE elements when conducting research, including* ***reporting on socio-economic considerations and regional disparities***” | 47 |
| **Technical Revisions**   1. Revise for technical errors, including consistent formatting and explanation of abbreviations.    - Reviewer 2, first comment (revise technical mistakes, e.g. abbreviations under tables) | **Technical Revisions**  Thank you for pointing this out. We have reviewed the tables and figures, and included legends were appropriate (Refer Table 2, Table 4 and Figure 2) |  |
| 2. Ensure that references are standardized and adhere to the required format.    - Reviewer 1, Suggestion 8 (standardise the format of references) | Thank you for identifying this error. We have now updated the references list, and adjusted numbers accordingly. | Throughout document |
| ***Note**: Reviewer 1 and 2 comments noted in blue and green text; we did not receive any comments from Reviewer 3 for changes | | |
